# Detection of *Ureaplasma* Biovars and Subtyping of *Ureaplasma parvum* among Women Referring to a University Hospital in Morocco

**DOI:** 10.1155/2020/7286820

**Published:** 2020-06-08

**Authors:** Safae Karim, Chahrazed Bouchikhi, Abdelaziz Banani, Hinde E. L. Fatemi, Tiatou Souho, Sanaa Erraghay, Bahia Bennani

**Affiliations:** ^1^Laboratoire de Microbiologie et de Biologie Moléculaire, Faculté de Médecine et de Pharmacie de Fès (FMPF), Université Sidi Mohammed Ben Abdellah (USMBA), Fès, Morocco; ^2^Service de Gynécologie CHU Hassan II de Fès, Morocco; ^3^Equipe Micro-organismes, Génomique et Facteurs Oncogènes, Laboratoire de Pathologie Humaine, Biomédecine et Environnement, FMPF, USMBA, Fès, Morocco; ^4^Equipe Anatomo-Pathology, Laboratoire Central d'Analyse Médicale, CHU Hassan II, Fès, Morocco

## Abstract

**Objectives:**

The aim of this study was to determine the prevalence of *Ureaplasma* biovars and *Ureaplasma parvum* (*U. parvum*) serovars, their associated risk factors, and genital STI-related symptoms.

**Methods:**

DNA obtained from cervical samples of 1053 women attending the department of Obstetrics and Gynecology and the laboratory of pathological anatomy of Hassan II university hospital of Fez, Morocco, was used to detect *Ureaplasma* biovars (*U. urealyticum* and *U. parvum*) and to subtype *U. parvum* by polymerase chain reaction (PCR).

**Results:**

Of the 1053 women examined, 25.4% (268/1053) were *Ureaplasma* positives. The rates of *U. urealyticum* and *U. parvum* were 12.1% (128/1053) and 7% (74/1053), respectively, and the copresence of these biovars was noted in 6.3% (66/1053) cases. The *U. parvum* subtyping revealed a predominance of the serovar 3/14 (61.4%). The association of demographics variables with *Ureaplasma* biovars was studied and shows that the age (“<30” years) seems to be a risk factor of *Ureaplasma* spp. and *U. urealyticum* carriage (OR 1.729, 95% CI [1.113-2.687] and OR 1.848, 95% CI [1.026-3.330], respectively). There was no difference in the prevalence of *Ureaplasma* type regarding symptoms. However, a significant association was found between *U. parvum* serovar 1 and infertility (*P* = 0.011).

**Conclusion:**

This first study conducted in Morocco provides an idea on *Ureaplasma* biovars and *U. parvum* serovars circulating in this region, their associated risk factors, and genital STI-related symptoms. Therefore, further studies are required to clarify and confirm the pathogenic role of these *Ureaplasma* species.

## 1. Introduction

Urogenital *Ureaplasma* belongs to the normal commensal flora of the human genital tract [1, 2]. However, it can be pathogenic when its bacterial load is ≥10^4^ organisms per ml (infectious dose). This rate is commonly accepted as a burden indicating an infection that should be treated [2, 3]. In fact, it can be associated with many gynaecological or obstetric pathologies such as nongonococcal urethritis, pelvic inflammatory disease, premature birth or late abortion, and infertility [4].

Human *Ureaplasma* spp. include two human pathogen species: *Ureaplasma urealyticum* (*U. urealyticum*) (biovar 2) and *Ureaplasma parvum* (*U. parvum*) (biovar 1). The specific identification of each species is based on molecular methods [5], and some studies shows that *U. parvum* is more common than the most pathogenic *U. urealyticum* [6]. The colonization rate in healthy women is about 18-87% for *U. parvum* and about 6-10% for *U. urealyticum* [7].

Based on biochemical and genetic characteristics [5], *U. parvum* (parvo biovar) is divided on four serovars (1, 3, 6, and 14) and *U. urealyticum* (biovar T960T) is separated on three subtypes. Subtype 1 includes serovars 2, 5, 8, and 9; subtype 2 comprises serovars 4, 10, 12, and 13; and subtype 3 contains serovars 7 and 11. A Polishian study showed that infection of the upper genitourinary tract with *U. parvum* is more common in infertile women than in fertile women [2]. Several studies suggest that the pathogenicity of *Ureaplasma* may be serotype-dependent, and others have shown that some serotypes are more frequently associated with syndromes than others [8–10]. *U. parvum* serovar 3 is the most frequently detected in infertile women and men, and some studies have revealed that the infiltration of *U. parvum* serovars 1, 3, and 6 caused morphological changes of the external genitalia in female mice, which can cause disorders of the superior genital tract that can lead to infertility [11]. However, *U. parvum* serovar 6 is the second most prevalent (in both women and men) and is associated with premature birth [12–14]. It is the leading cause of death among children under five years in developing countries [15].

In Morocco, there is no information about *Ureaplasma* biovars prevalence, serovars distribution of *U. parvum*, and their association with the risk factors and genital sexually transmitted infection- (STI-) related symptoms.

The present study aims at determining the prevalence of *Ureaplasma* biovars and *U. parvum* serovars, their associated risk factors, and genital STI- related symptoms.

## 2. Materiel and Methods

### 2.1. Patients and Sampling

A prospective study was conducted from 2013 to 2015 among women attending the Department of Obstetrics and Gynecology and the Laboratory of Pathological Anatomy of Hassan II University Hospital of Fez, Morocco, to determine the prevalence of bacterial STI and to characterize species [16]. All collected cervical samples were used to determine the prevalence of *Ureaplasma* biovars and to subtype *U. parvum*. All demographics and clinical data of the patients are available. Patients were divided in two groups, asymptomatic women who came to the gynecological examination because of a routine check-up and symptomatic women who had at least one of the following symptoms: leucorrhoea, pelvic pain/dyspareunia, pruritus, menorrhagia, metrorrhagia, or dysuria.

### 2.2. Ethics

The study was approved by the Institutional Review Board of Fez, Morocco (No. 02/15), and a written consent was obtained from all women.

### 2.3. *Ureaplasma* Biovars Detection and *U. parvum* Subtyping

*U. urealyticum* and *U. parvum* were detected in cervical samples by PCR using UreaA-B (F and R) and UMS-57/UMA222 primers, respectively, as described previously [5, 17]. The samples that were *U. parvum* positives were further typed, and their serovars were determined using a previously described PCR targeting the *mba* (multiband antigen) gene [5]. The specific primer pairs used as well as the size of the generated products were described in Table 1.

### 2.4. Statistical Analysis

Statistical analysis was performed using the SPSS (version 20) software. The different correlations were made using the chi-squared or Fisher's exact tests. The multivariate analysis was carried by binary logistic regression to determine the risk factors including all the variables with *P* ≤ 0.20 in the initial model. The results were expressed as odds ratio (OR), 95% confidence intervals (CIs), and *P* values. In all tests, a *P* value < 0.05 was considered as significant.

## 3. Results

### 3.1. Description of the Study Population

A total of 1053 patients were included in this study. The recruited participants were aged between 18 and 85 years (median age 42 years). Among these women, 29% (302/1053) are menopaused and 19% (197/1053) are pregnant. Of the 1053 women enrolled in the study, 39% (409/1053) were presented genital STI-related symptoms. The most common symptoms were leucorrhoea (32.3%; 132/409), pelvic pain/dyspareunia (26.6%; 109/409), pruritus (19.3%; 79/409), metrorrhagia (14%; 57/409), dysuria (4.6%; 19/409), and menorrhagia (3.2%; 13/409). Moreover, more than half (61%) (644/1053) of the participants had no genital STI-related symptoms. The socio-demographics and clinical characteristics of the study population are presented in Table 2.

### 3.2. Prevalence and Distribution of *Ureaplasma* Biovars and *U. parvum* Serovars

Of the 1053 women examined, 25.4% (268/1053) were *Ureaplasma* positives. The rates of *U. urealyticum* and *U. parvum* were 12.1% (128/1053) and 7% (74/1053), respectively, and both biovars were present in 6.3% (66/1053) cases. Considering the mixed infection/colonization, the prevalence of *U. urealyticum* was 18% (194/1053), and that of *U. parvum* was 13% (140/1053).

**Figure 1:**
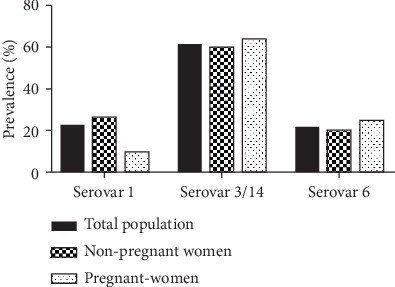
Distribution of *U. parvum* serovars in the study population.

The *U. parvum* typing shows that the serovar 3/14 was the most frequent (80/140, 57.1%), followed by the serovar 1 and the serovar 6 (20%, 28/140 and 17.9%, 25/140, respectively). This distribution was different when considering the pregnant women only. Thus, the serovar 3/14 is still the most predominant (64.5%) followed by serovar 6 (25.8%) and then the serovar 1 (9.7%) (Figure 1).

The presence of multiple serovars was noted in seven cases 5% (7/140) with a predominance of two serovars in six cases 4.3% (6/140) [Two cases (serovar 1 and serovar 3/14), three cases (serovar 3/14 and serovar 6), and one case (serovar 1 and serovar 6)] and a single case of triple serovar 0.7% (1/40). These coinfection/colonization serovars were detected only in nonpregnant women.

### 3.3. Risk Factors Associated with *Ureaplasma* Biovars and *U. parvum* Serovars

To determine the risk factors associated with *Ureaplasma* biovars and *U. parvum* serovars, a univariate analysis was performed by excluding the mixed cases of *U. urealyticum*/*U. parvum* (*N* = 66) and *U. parvum* serovars (*N* = 7).

Variables used for this analysis include socio-demographic factors, medical history, and sexual behaviour. The results of this analysis are presented in Table 3.

**Figure 2:**
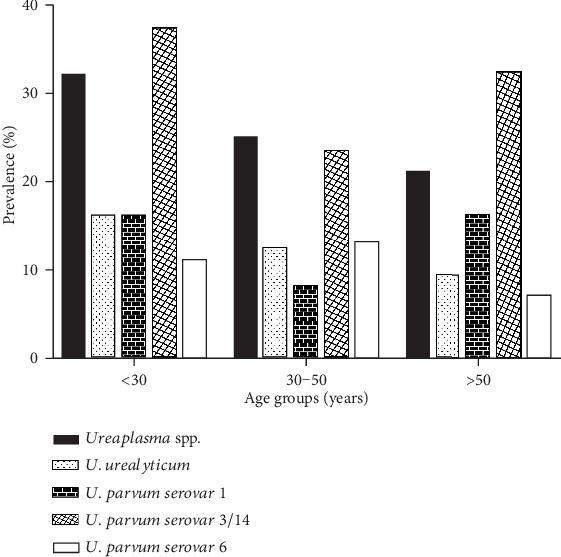
Prevalence of *Ureaplasma* biovars and *U. parvum* serovars according to participant's age.

The correlation of *Ureaplasma* biovars and *U. parvum* serovars to the age shows that *Ureaplasma* spp., *U. urealyticum*, and *U. parvum* serovar 3/14 had a peak of prevalence in women “<30” years-old. However, *U. parvum* serovar 6 was found maximally in the “30-50” years-old women and *U. parvum* serovar 1was more frequent in the “>50” years-old age group (Figure 2). Thus, the univariate analysis shows a significant association between *Ureaplasma* and “<30” years of age (*P* = 0.048) (Table 3).

All the variables with *P* value ≤ 0.2 were used in a multivariate analysis, and the results are presented in Table 4. Age (“<30” years) seems to be a risk factor of *Ureaplasma* spp. and *U. urealyticum* carriage (OR 1.729, 95% CI [1.113-2.687] and OR 1.848, 95% CI [1.026-3.330], respectively).

### 3.4. Correlation between *Ureaplasma* Biovars and *U. parvum* Serovars and Genital STI-Related Symptoms

In order to study the correlation between genital STIs-related symptoms and *Ureaplasma* biovars, a statistical analysis was performed. For this analysis, *Ureaplasma* negative cases and the coinfection of *Ureaplasma* spp. with other STI (Human papillomavirus, *Chlamydia trachomatis*, *Neisseria gonororhea*, *Mycoplasma genitalium*, and *Mycoplasma hominis*) cases were excluded. Therefore, only 87 *Ureaplasma* cases were considered (45 and 42 cases of *U. urealyticum* and *U. parvum*, respectively). The results of correlations were presented in Table 5. Thus, 36 women were with genital STI-related symptoms vs. 51 of asymptomatic women, and 5 women were infertile vs. 82 of fertile women. *U. urealyticum* and *U. parvum* were equally distributed among symptomatic and asymptomatic patients. However, a higher frequency of *U. parvum* was observed in infertile women (Table 5).

To determine the correlation between the presence of genital STI-related symptoms and *U. parvum* serovars, cases with multiples serovars (*N* = 7) were excluded. The results were presented in Table 6. The results show a significant association between *U. parvum* serovar 1 and infertility (*P* = 0.011) (Table 6).

## 4. Discussion

*Ureaplasma* spp. mainly resides on the mucous surfaces of the urogenital tract in adults or in the respiratory tract of infants [18]. These species can be responsible of nongonococcal urethritis and pregnancy complications. Their incidence is higher in women compared to men [19], and their colonization was related to age, low socio-economic level, multiplicity of sexual partners, ethnicity, and oral contraceptives uses [20].

To the best of our knowledge, this is the first study conducted in Morocco with the aim at determining the distribution, the prevalence of *Ureaplasma* biovars and the circulating *U. parvum* serovars, and their association with risk factors and genital STI-related symptoms.

The present study shows that 25% of women harbored *Ureaplasma* spp. This rate is slightly similar to that obtained in an Italian study (23%) [21], but lower than the rate obtained in Croatian study (34%) [3] (Table 7). Nevertheless, in the Italian study, the obtained prevalence represents only cases of *Ureaplasma* culture-positive with a bacterial load of ≥10^4^ CFU (infection cases). This can explain the low rate obtained compared to our results, where *U. u*realyticum detection has been made directly by PCR without bacterial quantification.

Regarding *Ureaplasma* biovars, our results showed a predominance of *U. urealyticum* (18%; 194/1053) compared to *U. parvum* (13%; 140/1053) (considering the mixed infection/colonization). This distribution seems to be inversed compared to that reported on other geographical area such as in Italia and Croatia [3, 21]. The *U. parvum* subtyping results show that *U. parvum* serovar 3/14 was the most prevalent followed by serovars 1 and 6, respectively. Similar distribution was observed in the Italian study even if the rates are different [21] (Table 7). The study population and geographic location can explain this difference.

In nonpregnant women group and independently of their fertility status, the prevalence of *U. urealyticum* and *U. parvum* was 11.6% (99/856) and 6.8% (58/856), respectively (excluding cases of mixed detection). These distribution and prevalence are different from that reported in a Brazilian study, which was marked by high prevalence of *U. parvum* (60.6%) [22] (Table 7). The difference is notably observed at *U. parvum* serovar distribution level and was marked [22], in comparison with our result, by the lowest rate of serovar 1 and higher cases of coinfection/colonization [22] (Table 7). This difference may be related to geographic area, sample size, and the sexual activity.

In pregnant women group, the *U. urealyticum* is more predominant than *U. parvum* (14.7% (29/197) vs. 8.1% (16/197)), respectively (and considering simple infection/colonization only), inversely to results obtained on Australian women [14] (Table 7). The difference was also obtained in *U. parvum* type distribution that even if the serovar 3/14 is predominant in both populations, the serovar 1 is largely prevalent than serovar 6 in the Australian study [5] (Table 7). Nevertheless, our serovars distribution is similar to that recently obtained in an American and another Australian studies (where serovars 3 and 6 are the most frequent) [14, 23] (Table 7). This can raises the question of epidemiological evolution or changes of species distribution over the time. The fact that in these later studies, serovar 6 was significantly associated with preterm delivery [14, 23] lets us suppose that pregnant women of our series, carrying serovar 6 (25.8%), were probably at risk of premature delivery. Thus, it is necessary to supervise their pregnancy progress in order to verify this hypothesis and to strengthen screening programs for *Ureaplasma* to prevent complications.

In our study, the presence of multiple *U. parvum* serovars was not detected in the group of pregnant women while it was reported in 8% and 2.8% of cases in Australian studies [5, 14] (Table 7). This difference may be related to the geographic location and sexual behaviors of women as well as that of their partners.

The correlation between *U. parvum* serovars and age has been studied and the results show that high levels of *U. parvum* serovar 3/14 and serovar 6 were mainly obtained in young women “≤50 years” (which can be related to sexual activity), while the *U. parvum s*erovar 1 was more common among women aged “>50” years. This can be related to the hormonal status (menopause) of these women as reported by Iwasaka et al. who described the presence of *Ureaplasma* spp. in vaginal flora of 25% of postmenopausal women [24]. In fact, menopause may be associated with vaginal atrophy, thinning of the vaginal wall, dryness, and changes in pH due to a lack of estrogen, factors that may affect sexual function, and promotes infection/colonization [25].

Using logistic regression models, a significant association was obtained between the age group “<30 years” and *Ureaplasma* spp. (*P* = 0.048). These results are not surprising and confirm the results of previous studies [26, 27] as well as the fact that genital mycoplasmas are linked to sexual activity. In our series, most of the recruited patients declared that they had only one sexual partner during their life (their husband) which is related to socio-cultural and religious context. Thus, the association between sexual behavior and *Ureaplasma* biovars cannot be performed.

The correlation between *Ureaplasma biovars* and genital STI-related symptoms shows no association neither for *U. parvum* nor for *U. urealyticum*. These results are consistent with those of other studies conducted in Australia, Slovenia, and Croatia [3, 28, 29]. However, an Italian study reported that *U. urealyticum* and *U. parvum* serovar 3/14 were significantly associated with symptomatic patients [21]. In our study, a significant association between *U. parvum* serovar 1 and infertility was obtained (*P* = 0.011). To confirm this data, the study of larger population and refinement of *Ureaplasma* spp. molecular characterization will be of interest.

There were some limitations of the present study: firstly, the patients participating in this study may not be truly representative of the Moroccan population; in other words, the data derived from this region may not reflect the situation of other geographical regions and studies in the other Moroccan regions are required. Secondly, following the successive recruitment of patients, the sample size in the groups of asymptomatic and symptomatic women was different. Likewise, the cultivation and quantification of *Ureaplasma* spp. were not carried out. Therefore, simple colonization cannot be distinguished from infection. However, our main objective was to determine the prevalence of *Ureaplasma* biovars and *U. parvum* serovars, their associated risk factors, and genital STI-related symptoms.

## 5. Conclusion

In this study, the distribution and prevalence of *Ureaplasma* biovars and each of the *U. parvum* serovars have been determined. Thus, the results showed the predominance of *U. urealyticum* compared to *U. parvum*. Moreover, the *U. parvum* subtyping revealed that the serovar 3/14 is the most prevalent followed by serovars 1 and 6 and a significant association between serovar 1 and infertility. This lets suggest the need of testing this species in the infertility cases.

The obtained results provide an idea on *Ureaplasma* biovars and *U. parvum* serovars distribution in Morocco and especially in Fez region area, their associated risk factors, and genital STI-related symptoms. Further studies are needed to clarify and confirm the pathogenic role of these species.

## Figures and Tables

**Table 1:** Primers used in this study.

Species	Primer (forward and reverse)	Sequence (5′—3′)	Size (bp)	Reference
*U. urealyticum*	ureA-B	GAA ACG ACG TCC ATA AGC AAC T	423	[17]
		GCA ATC TGC TCG TGA AGT ATT AC		
*U. parvum*	UMS-57	(T/C)AA ATC TTA GTG TTC ATA TTT TTT AC	326/327	[5]
	UMA222	GTA AGT GCA GCA TTA AAT TCA ATG		
*U. parvum* serovar 1	UMS83	TACTGATAGAAATTATGTAAGATTGC	398	
	UMA269′	CCAAATGACCTTTTGTAACTAGAT		
*U. parvum* serovar 6	UMS54	CTTAGTGTTCATATTTTTTACTAG	369	
	UMA269′	CCAAATGACCTTTTGTAACTAGAT		
*U. parvum* serovar 3/14	UMS125	GTATTTGCAATCTTTATATGTTTTCG	442	
	UMA269	CTAAATGACCTTTTTCAAGTGTAC		

**Table 2:** The socio-demographics and clinical characteristics of the study population.

Variable	*N*	%
Area, *n* = 1035		
Rural	212	20.5
Urban	823	79.5
Age (years), *n* = 1047		
<30	167	16
30-50	624	59.5
>50	256	24.5
Menopause, *n* = 1033		
No	731	70.8
Yes	302	29.2
Education level, *n* = 1038		
Illiterate	657	63.3
Literate	381	36.7
Number of pregnancies, *n* = 1037		
≤4	724	69.8
>4	313	30.2
Parity, *n* = 1038		
≤4	832	80.2
>4	206	19.8
Passive smoking, *n* = 1031		
No	785	76.1
Yes	246	23.9
Oral contraception, *n* = 1030		
No	722	76.1
Yes	246	23.9
Age at 1st sexual intercourse (years), *n* = 1031		
≤20	656	63.6
>20	375	36.4
Number of lifetime sexual partners, *n* = 1028		
1	974	94.7
≥1	54	5.3
Pregnancy status		
No	856	81
Yes	197	19
Presence of genital STI-related symptoms		
No	644	61
Yes	409	39
Infertility		
No	1006	95.5
Yes	47	4.5

**Table 3:** Prevalence of *Ureaplasma* biovars according to different variables.

Characteristics	Number total of participants	*U. urealyticum* (positive) (*N* = 128)	*U. parvum* (positive) (*N* = 74)	*U. urealyticum*/*U. parvum* (positive) (*N* = 66)	*Ureaplasma* spp. (positive) (*N* = 268)
		*n* (%)			
Area					
Rural	212	26 (12.3)	14 (6.6)	18 (8.5)	58 (27.4)
Urban	823	100 (12.2)	59 (7.2)	48 (5.8)	207 (25.2)
*P*		0.96	0.774	0.158	0.512
Age group (years)					
<30	167	27 (16.2)	11 (6.6)	16 (9.6)	54 (32.3)
30-50	624	76 (12.2)	47 (7.5)	35 (5.6)	158 (25.3)
>50	254	24 (9.4)	16 (6.3)	15 (5.9)	55 (21.7)
*P*		0.119	0.782	0.165	0.048
Menopause					
No	731	97 (13.3)	49 (6.7)	49 (6.7)	195 (26.7)
Yes	302	29 (9.6)	22 (7.3)	17 (5.6)	68 (22.5)
*P*		0.101	0.737	0.521	0.163
Education level					
Illiterate	657	83 (12.6)	44 (6.7)	42 (6.4)	169 (25.7)
Literate	381	43 (11.3)	29 (7.6)	24 (6.3)	96 (25.2)
*P*		0.522	0.579	0.953	0.851
Number of pregnancies					
≤4	724	94 (13.0)	45 (6.2)	50 (6.9)	189 (26.1)
>4	313	32 (10.2)	28 (8.9)	16 (5.1)	76 (24.3)
*P*		0.212	0.115	0.277	0.536
Parity					
≤4	832	104 (12.5)	57 (6.9)	56 (6.7)	217 (26.1)
>4	206	22 (10.7)	16 (7.8)	10 (4.9)	48 (23.3)
*P*		0.474	0.645	0.323	0.413
Passive smoking					
No	785	94 (12.0)	58 (7.4)	56 (7.1)	208 (26.5)
Yes	246	31 (12.6)	15 (6.1)	10 (4.1)	56 (22.8)
*P*		0.793	0.491	0.086	0.242
Oral contraception					
No	722	89 (12.3)	52 (7.2)	48 (6.6)	189 (26.2)
Yes	308	37 (12.0)	21 (6.8)	18 (5.8)	76 (24.7)
*P*		0.888	0.826	0.630	0.614
Age at 1st sexual intercourse (years)					
≤20	656	84 (12.8)	47 (7.2)	36 (5.5)	167 (25.5)
>20	375	42 (11.2)	26 (6.9)	30 (8.0)	98 (26.1)
*P*		0.449	0889	0.113	0.811
Number of lifetime sexual partners					
1	974	122 (12.5)	68 (7.0)	60 (6.2)	250 (25.7)
≥1	54	4 (7.4)	5 (9.3)	6 (11.1)	15 (27.8)
*P*		0.264	0.336	0.126	0.730

*P* value, *χ*^2^, or Fisher's exact test.

**Table 4:** Risk factors associated with *Ureaplasma* biovars (multivariate analysis).

	Variables		Odds ratio	95% CI	*P* value
*Ureaplasma* spp.	Age (years)	<30	1.729	1.113-2.687	0.015
		30-50	1.227	0.865-1.739	0.251
		>50	1	—	—
*U. urealyticum*	Age (years)	<30	1.848	1.026-3.330	0.041
		30-50	1.329	0.819-2.157	0.249
		>50	1	—	—

**Table 5:** Correlation between *Ureaplasma* biovars with clinical symptomatology and infertility.

	*Ureaplasma* biovars		
	*U. urealyticum*, *N* = 45	*U. parvum*, *N* = 42	*P* value
	*n* (%)		
Genital STI-related symptoms			
Yes, *N* = 36	19 (52.8)	17 (47.2)	0.869
No, *N* = 51	26 (51.0)	25 (49.0)	
Infertility			
Yes, *N* = 5	2 (40.0)	3 (60.0)	0.591
No, *N* = 82	43 (52.4)	39 (47.6)	

*P* value, *χ*^2^, or Fisher's exact test.

**Table 6:** Correlation between *U. parvum* serovars with clinical symptomatology and infertility.

	*U. parvum* serovars		
	Serovar 1, *N* = 6	Serovar 3/14, *N* = 30	Serovar 6, *N* = 3
	*n* (%)		
Genital STI-related symptoms			
Yes, *N* = 17	1 (5.9)	15 (88.2)	1 (5.9)
No, *N* = 22	5 (22.7)	15 (68.2)	2 (9.1)
*P* value	0.154	0.146	0.713
Infertility			
Yes, *N* = 3	2 (66.7)	1 (33.3)	0 (0)
No, *N* = 36	4 (11.1)	29 (80.6)	3 (8.3)
*P* value	0.011	0.066	0.607

*P* value, *χ*^2^, or Fisher's exact test.

**Table 7:** *Ureaplasma* biovars and *U. parvum* serovars according to geographical area.

Study, region	Study group	Prevalence % (*n*/*N*)							
		*Ureaplasma* spp.	UU	UP	UU/UP	SV UP			
						SV 1	SV 3/14	SV 6	Multiple SV
Our study, Morocco	1053: P and NP women	25.4 (268/1053)	12.1 (128/1053)	7 (74/1053)	6.3 (66/1053)	20 (28/140)	57.1 (80/140)	17.9 (25/140)	5 (7/140)
[21]; Italy	806: P and NP women	23 (186/806)	14 (22/158^∗^)	86 (136/158^∗^)	None	37 (50/136)	39 (53/136)	24 (33/136)	None
[3]; Croatia	1370: P and NP women	34.4 (471/1370)	7.4 (18/244^×^)	92.6 (226/244^×^)	None	ND	ND	ND	ND
Our study, Morocco	856: NP women	24.3 (208/856)	11.6 (99/856)	6.8 (58/856)	6 (51/856)	26.6 29/109	60.6 (66/109)	15.6 (22/109)	6.4 (7/109)
[22]; Brazil	302: sexually active NP women	76.2^≠^ (230/302)	16.6 (50/302)	60.6 (183/302)	4.6 (14/302)	23.6 (37/156^”^)	39.5 (62/156^”^)	40.8 (64/156^”^)	11.5 (18/156^”^)
Our study; Morocco	197: P women	30.5 (60/197)	14.7 (29/197)	8.1 (16/197)	7.6 (15/197)	9.7 (3/31)	64.5 (20/31)	25.8 (8/31)	None
[14]; Australia	191: P women	48 (91/191)	13 (25/191)	39 (74/191)	ND	15.3 (11/72^#)^	SV3: 37.5 (27/72^#^) SV14: 0 (0/72^#^)	43 (31/72^#^) SV6.1: 1.4 (1/72^#^)	2.8 (2/72^#^)
[5]; Australia	78: U from vaginal swabs of P women	78 *Ureaplasma* positives	19.2 (15/78)	79.5 (62/78)	1.3 (1/78)	27 (17/63)	49 (31/63)	16 (10/63)	8 (5/63)
[23]; United States	169: U from vaginal swabs of P women	169 *Ureaplasma* positives	14 (24/169)	81 (137/169)	4 (7/169^±^)	23 (33/144)	SV3: 59.7 (86/144) SV14: 4.2 (6/144)	28.5 (41/144)	ND

P: pregnant; NP: nonpregnant; UU: *Ureaplasma urealyticum*; UP: *Ureaplasma parvum*; SV: serovar; ND: not determined. ^∗^Total culture positive only for *Ureaplasma urealyticum.*^×^Only 244 samples were successfully genotyped to UU and UP. ^≠^76.2% (230/302) of the women studied were colonized by Mollicutes. ^”^27 *U. parvum* positives were negative for all four known *U. parvum* serovars. ^#^No amplification 1% (2/72). ^±^Negative *Ureaplasma* spp. 1% (1/169).

## Data Availability

The data used to support the findings of this study are available from the corresponding author upon request.
